# The Crosstalk between HepG2 and HMC-III Cells: In Vitro Modulation of Gene Expression with Conditioned Media

**DOI:** 10.3390/ijms232214443

**Published:** 2022-11-21

**Authors:** Prashant Koshal, Ilenia Matera, Vittorio Abruzzese, Angela Ostuni, Faustino Bisaccia

**Affiliations:** Department of Sciences, University of Basilicata, 85100 Potenza, Italy

**Keywords:** microglia, HepG2 cells, endoplasmic reticulum stress, autophagy, mitophagy, inflammation

## Abstract

Epidemiological studies have postulated an inverse correlation between developing cancer and neurodegeneration. It is known that the secretome plays a vital role in cell–cell communication in health and disease; the microglia is the resident macrophage of the central nervous system which maintains neuronal integrity by adapting as the microenvironment changes. The present study aimed to identify, in a cell model, biomarkers that link neurodegenerative diseases to cancer or vice versa. Real-time PCR and western blot analysis were used to characterize the effects on gene and protein expression of human hepatoblastoma (HepG2) and human microglia (HMC-III) cells after exchanging part of their conditioned medium. Biomarkers of the endoplasmic reticulum, and mitophagy and inflammatory processes were evaluated. In both cell types, we observed the activation of cytoprotective mechanisms against any potential pro-oxidant or pro-inflammatory signals present in secretomes. In contrast, HepG2 but not HMC-III cells seem to trigger autophagic processes following treatment with conditioned medium of microglia, thus suggesting a cell-specific adaptive response.

## 1. Introduction

The inverse correlation between developing cancer and neurodegeneration has been reported in many epidemiological studies, thus suggesting that cancer survivors are less likely to suffer from a neurodegenerative disorder or vice versa [1,2,3,4]. In fact, many case-control and cohort studies have revealed a decreased risk of many cancers in survivors of Alzheimer’s disease (AD) and Parkinson’s disease (PD). However, an exceptionally increased risk of some types of cancer such as melanoma and breast cancer has been reported [5,6,7,8]. A recent study, conducted by Ibáñez et al., showed that up-regulated transcripts in cancer are down-regulated in central nervous system (CNS) diseases or vice versa. Moreover, it is hypothesized that this inverse correlation is driven by molecular processes common to CNS disorders and cancers and that are deregulated in opposite directions [9]. The inverse correlation between cancer and neurodegeneration might be possible due to sharing the same molecular and genetic pathways for two different biological processes, where cancer cells continuously proliferate and neurons undergo post-mitotic cell death [10,11,12,13,14]. A better understanding of cellular factors and signals involved in the regulation of these age-related diseases is relevant and can have important clinical implications. The identification of prognostic markers in cancer and neurodegeneration might open new therapeutic windows for both diseases [3,15]. In addition, aging, which is defined as the loss of cellular physiological functions, is an important factor associated with the development of cancer and neurodegeneration [16,17,18], although the exact mechanism that leads to aging and can thus provide a link between these two diseases, is not fully understood [19,20].

Alteration in the integrity of the blood–brain barrier (BBB) leads to the development of chronic neurological disorders such as Alzheimer’s disease (AD), Parkinson’s disease (PD), multiple sclerosis (MS), acute and chronic cerebral ischemia, trauma, and Amyotrophic lateral sclerosis (ALS); this is due to sustained glial cell activation, increased infiltration of peripheral immune cells and plasma proteins, and ionic dysfunction [21,22,23]. Moreover, studies revealed that alteration in the metabolic, autocrine, and paracrine functions are capable of modifying cellular characteristics, which can lead to the development of various chronic diseases, such as cancer and neurodegeneration [24,25,26,27,28,29]. In addition, it has been suggested that conditioning media, which contain cell secretomes, are beneficial for studying cell–cell communication in normal and disease conditions [30,31]. These observations suggest that the inverse correlation between tumors and neurodegeneration may be the consequence of a direct action of the tumor cell secretome on microglia, the resident macrophage of the CNS which maintains the neuronal integrity in health and disease [32,33], and adapts as the microenvironment changes. In the resting stage, microglia produce neuroprotective factors to nourish the neurons under either physiological or pathological conditions. However, under chronic activation, this can lead to neuronal loss [34,35,36]. 

In the present study, with the aim to identify biomarkers which link neurodegenerative diseases to cancer or vice versa, the expression level of transcripts and proteins was evaluated in a model system consisting of human hepatoblastoma (HepG2) and human microglia (HMC-III) cells grown in mutually exchanged conditioned media.

## 2. Results

### 2.1. Effect of Conditioned Media on Viability of HepG2 and HMC-III Cells

**Figure 1:**
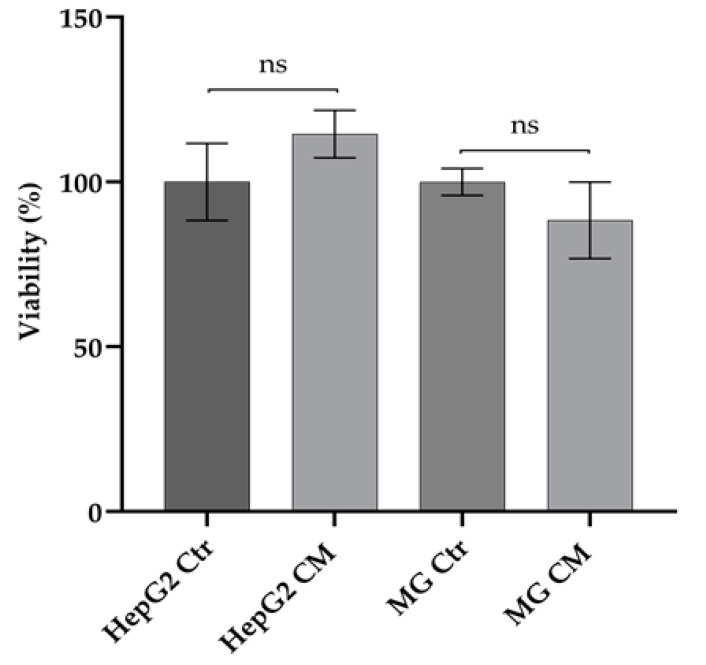
Effect of conditioned media on cell viability. Data represent the percentage cell viability of HepG2 and HMC-III cells grown for 48 h in conditioned (HepG2 CM and MG CM, respectively) and own-culture medium (HepG2 Ctr and MG Ctr, respectively). Standard error mean (SEM) was calculated using three replicates of three independent experiments. Statistical significance was assessed by using *t*-test. ns: not significant *p*-value.

An MTT assay was performed to verify the effect of conditioned media (CM) on cell viability (Figure 1). No significant difference was observed at up to 48 h; it was therefore chosen for further experiments.

### 2.2. Effect of Conditioned Media on the Expression Level of Endoplasmic Reticulum Stress Markers

**Figure 2:**
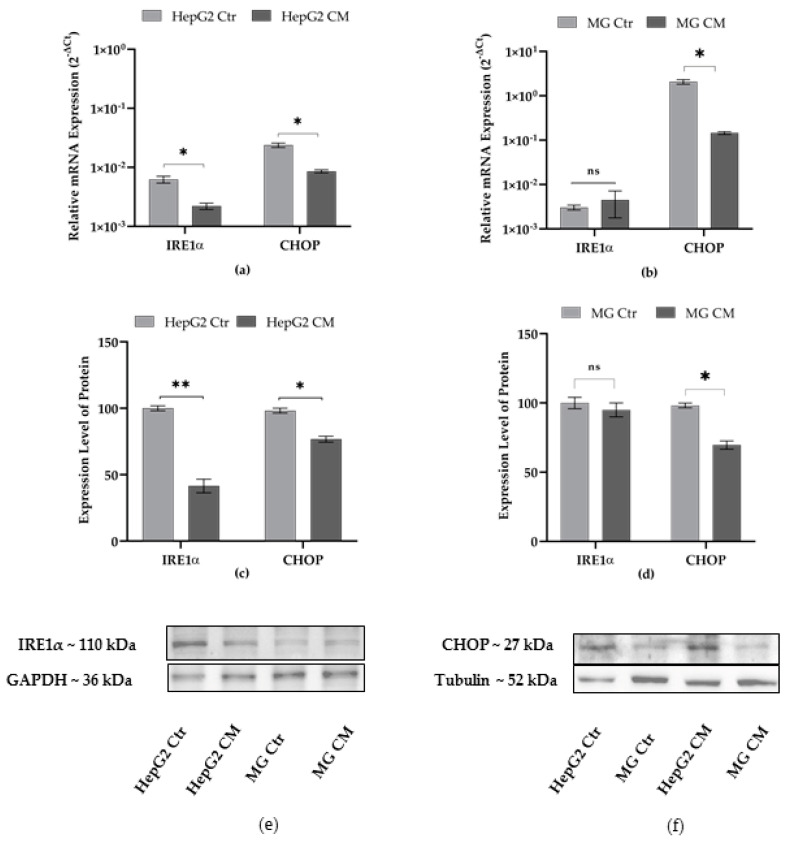
Effect of conditioned media on expression level of IRE1α and CHOP. Transcript levels in (**a**) HepG2 and (**b**) HMC-III (MG) cells are expressed as 2^−ΔCt^ and presented as the mean ± SEM of at least three different experiments. Protein expression level in (**c**) HepG2, (**d**) MG cells; (**e**,**f**) representative western blots. The protein levels were normalized with GAPDH or Tubulin content; then, for each cell type grown in conditioned medium (CM), data refer to the corresponding control cells grown in their own culture medium (Ctr), and set to 100%. Results are expressed as the mean ± the standard error of three independent experiments. Statistical analysis was performed by using *t*-test; * *p* < 0.05, ** *p* < 0.01; control (Ctr) cells vs. media conditioned treated (CM) cells; ns: not significant *p*-value.

It is widely accepted that endoplasmic reticulum (ER) stress plays an important role in cancer as well as in neurodegeneration [37]. However, in cancer cells, ER stress-mediated activation of unfolded protein responses (UPRs) has been found to support cancer cell survival and chemoresistance [37,38]. In neurodegenerative disorders, ER stress-mediated activation of UPR attempts to minimize the protein misfolding for cell survival, whereas sustained UPR activation leads to the apoptosis of neuronal cells [38,39]. In the present study, the expression levels of IRE1α and CHOP were evaluated after cell treatment with conditioned media (CM). The RNA and protein expression levels of IRE1α decreased in HepG2 but not in microglia cells (Figure 2a,b), whereas the mRNA and protein expression of CHOP decreased in both cell types (Figure 2c,d).

### 2.3. Effect of Conditioned Media on Mitophagy Markers

**Figure 3:**
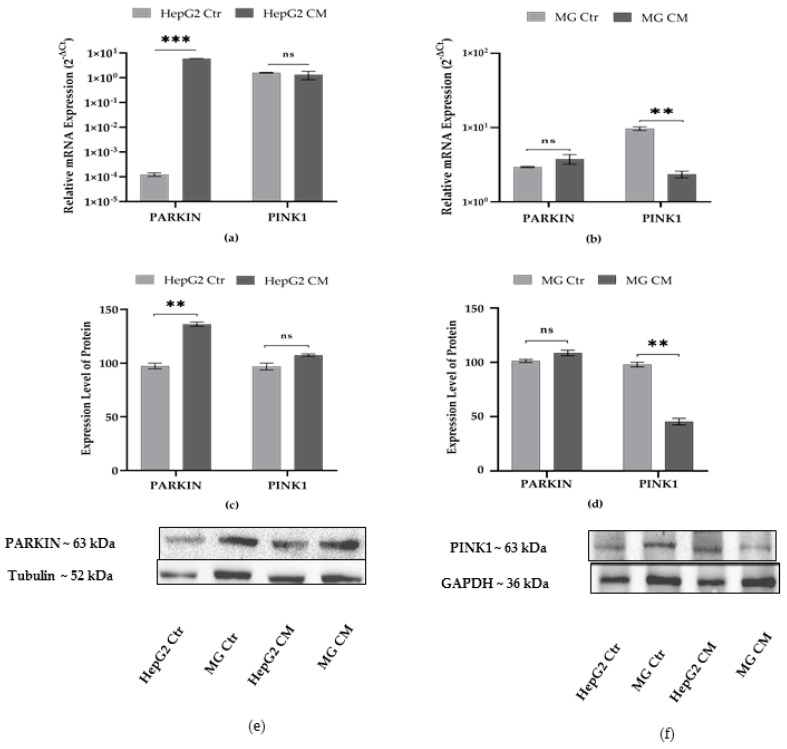
Effect of conditioned media on expression levels of PARKIN and PINK1. Transcript levels in (**a**) HepG2 and (**b**) HMC-III (MG) cells are expressed as 2^−ΔCt^ and presented as the mean ± SEM of at least three different experiments. The protein expression level in (**c**) HepG2, (**d**) MG cells; (**e**,**f**) are representative western blots. The protein levels were normalized with GAPDH or Tubulin content; then, for each cell type grown in conditioned medium (CM), data refer to the corresponding control cells grown in their own culture medium (Ctr), set to 100%. Results are expressed as the mean ± the standard error of three independent experiments. Statistical analysis was performed by using *t*-test; ** *p* < 0.01, *** *p* < 0.001, control (Ctr) cells vs. media conditioned treated (CM) cells; ns: not significant *p*-value.

Mitochondria play an important role in maintaining cellular homeostasis and their quality is determined by the RBR E3 ubiquitin–protein ligase (PARKIN) and PTEN-induced kinase 1 (PINK1) dependent pathway. Change in mitochondrial bioenergetics leads to the recruitment of PARKIN by mitochondrial PINK1, which direct the degradation of defective mitochondria by autophagosomes. Defects in the mitochondrial quality control machinery play a possible role in cancer progression and neurodegeneration [40,41,42,43]. In HepG2 cells grown in conditioned medium, the expression level of PARKIN transcripts increased but no changes were observed in PINK1; in contrast, PINK1 transcripts decreased in MG CM (Figure 3a,b). These results are also confirmed by protein analysis (Figure 3c,d).

### 2.4. Effect of Condition Media on Autophagy Markers

**Figure 4:**
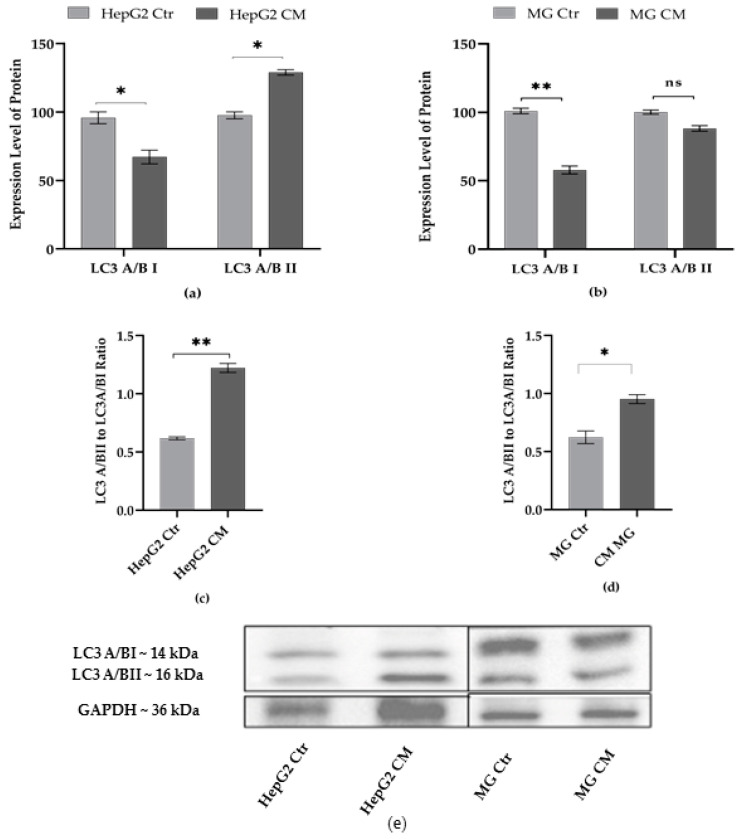
Effect of conditioned media on protein levels of LC3 A/B I and LC3 A/B II. The protein levels of (**a**) HepG2 and (**b**) HMC-III (MG) cells were normalized with GAPDH content; then, for each cell type grown in conditioned medium (CM), data refer to the corresponding control cells grown in their own culture medium (Ctr), set to 100%. (**c**,**d**) The LC3II/I ratio for each cell types. (**e**) Representative western blots. Densitometric analysis of the immunoreactive bands performed in three independent experiments and results are expressed as the mean ± the standard error. Statistical analysis was performed by using *t*-test; * *p* < 0.05, ** *p* < 0.01; control (Ctr) cells vs. media conditioned treated (CM) cells; ns: not significant *p*-value.

In the eukaryotic cell, autophagy is a catabolic process in which autophagosomes degrade cellular organelles or proteins to induce cellular homeostasis. The dysregulation of this machinery plays an important role in cancer progression and neurodegeneration [44,45]. The expression level of the autophagy markers in HepG2 and MG cells treated with the conditioned media has been evaluated (Figure 4a,b). The ratio LC3II/I, representing the autophagic rate, showed a higher value in HepG2 cells treated with conditioned media (Figure 4c) rather than in MG CM cells (Figure 4d), thus suggesting a greater activation of the autophagic process in hepatoblastoma cells.

### 2.5. Effect of Conditioned Media on Inflammatory Markers

Inflammation mediated by iNOS and NFkB plays an important role in the development and progression of cancer [46,47], microglial activation, and neurodegeneration due to neurotoxin or inflammatory damage [48,49]. Moreover, Nrf2 plays an important role in association with NFkB in cancer and neurodegeneration. Studies revealed that Nrf2 inhibits the activation of NFkB and leads to an anti-inflammatory effect [50,51]. TNFR2 plays an important role in tumor microenvironments, protects the tumor cell from immune surveillance [52,53], and in the microglial cell, leads to immunomodulatory, anti-inflammatory, and neuroprotective effects [54,55]. 

**Figure 5:**
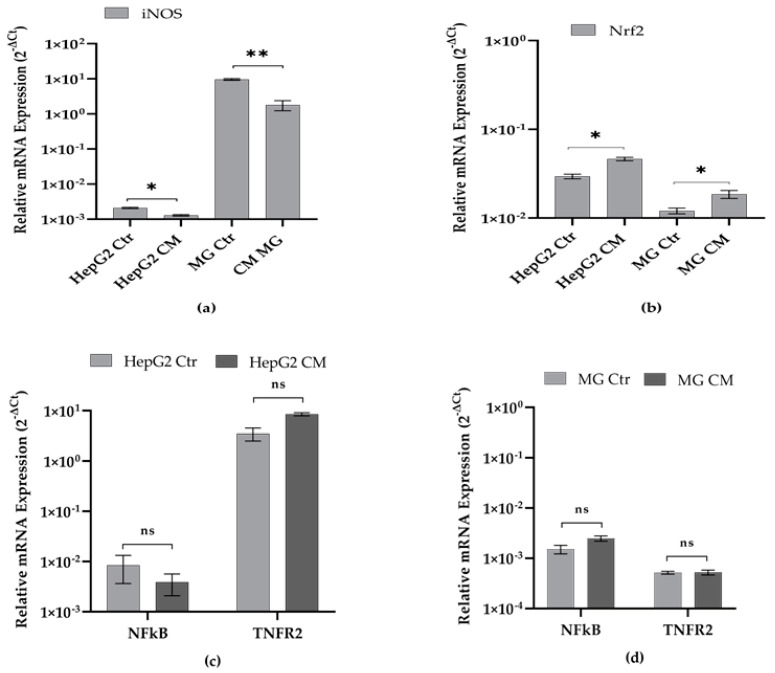
Effect of conditioned media on expression levels of iNOS, Nrf2, NFkB, and TNFR2. Transcript levels of (**a**) iNOS, **(b**) Nrf2, (**c**,**d**) NFkb and TNFR2 were evaluated in HepG2 and HMC-III cells grown for 48 h in conditioned (HepG2 CM and MG CM, respectively) and own-culture medium (HepG2 Ctr and MG Ctr, respectively). Results are expressed as 2^−ΔCt^ and presented as the mean ± SEM of at least three different experiments. Statistical analysis was performed by using *t*-test; * *p* < 0.05, ** *p* < 0.01; control (Ctr) cells vs. media-conditioned (CM) cells; ns: not significant *p*-value.

RT-PCR was performed to investigate the changing pattern of the above-mentioned genes. A decrease in iNOS (Figure 5a) and a slight increase in Nrf2 (Figure 5b) transcripts of HepG2 CM and MG CM cells were observed, whereas no significant difference was observed in the transcripts of NFkB and TNFR2 in both cell types after treatment with conditioned media (Figure 5c,d).

### 2.6. Effect of Conditioned Media on Cell Cycle Markers

**Figure 6:**
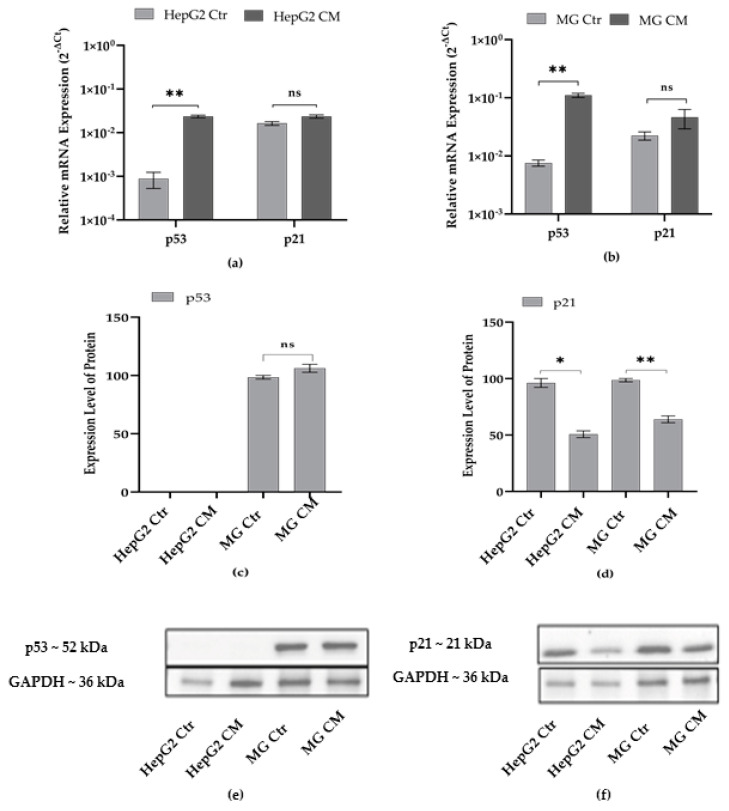
Effect of conditioned media on expression levels of p53 and p21. Transcript and protein expression levels were evaluated in HepG2 (**a**,**c**) and HMC-III cells (**b**,**d**) grown for 48 h in conditioned (HepG2 CM and MG CM, respectively) and own-culture medium (HepG2 Ctr and MG Ctr, respectively). Transcript levels are expressed as 2^−ΔCt^ and presented as the mean ± SEM of at least three different experiments. The protein levels were normalized with GAPDH; then, for each cell-type grown in conditioned medium (CM), data refer to the corresponding control cells grown in their own culture medium (Ctr), set to 100%. (**e**,**f**) Representative western blots. Results are expressed as the mean ± the standard error of three independent experiments. Statistical analysis was performed by using *t*-test; * *p* < 0.05, ** *p* < 0.01; control (Ctr) cells vs. media conditioned (CM) cells; ns: not significant *p*-value.

It is widely accepted that p53 plays an key role in tumor suppression in a p21-dependent manner. However, studies suggest that p53 is crucially involved in microglial pro-inflammatory response and neurodegeneration [56,57]. To investigate if the conditioned media mutually exchanged between the two cell types lead to any effect on the cell cycle, RT-PCR was performed. The transcript level of p53 was found to increase both in HepG2 CM (Figure 6a) and MG CM (Figure 6b) cells; conversely, the transcript level of p21 did not change. The Western blot analysis revealed no expression of p53 in HepG2 CM cells and no change in the level of expression in MG CM (Figure 6c). However, after growing in conditioned media, both the cell types showed a reduced expression of the p21 protein (Figure 6d).

## 3. Discussion

Cancer and neurodegeneration are chronic diseases which, with the exception of a very limited number of cases, have shown an inverse incidence in the same patient age groups. Although there is a partial overlap of the genes involved in the two diseases, the molecular mechanisms underlying the mutations and/or the alteration of their expression respond to opposite characteristics of the cells, namely proliferation and death from cancer and neuronal cells, respectively. However, a possible contribution of the cellular secretome to this “opposite” modulation of both genes and proteins in cancer and neuronal cells cannot be excluded. In order to verify this hypothesis, we have designed an experimental protocol (see Materials and Methods) based on the exchange of medium between HepG2 hepatoblastoma and HMC-III microglia cells. Genes, proteins, and pathways dysregulated in both cancer and neurodegenerative diseases have been explored. 

The effect of conditioned media on the ER stress markers was evaluated. It is clear that the ER stress-mediated activation of UPRs plays a crucial role in restoring cell physiology, whereas the prolonged activation of UPRs leads to metabolic changes as well as apoptotic cell death [58,59]. Studies revealed that tumor cells possess an adaptive phenomenon with activated UPRs, which provides a hospitable environment for tumor survival and progression, and even protects the cells from chemotherapeutic agents [60,61]. In contrast, sustained UPR activation is involved in neurodegeneration [62]. We found decreased transcript and protein expression levels of CHOP and IRE1α in HepG2 CM, whereas only CHOP was found to be decreased in MG CM (Figure 2) compared to their respective controls. Failure to activate the CHOP and IRE1α sensors, which play a critical role in the initiation of pro-apoptotic signals, suggests the lack of a sustained reticulum stress condition over time.

The cellular response to ER stress as an attempt to control protein homeostasis activates autophagy and mitophagy on PARKIN- and PINK1-dependent pathways. Under normal conditions, PINK1 is imported in mitochondria, cleaved, and then degraded by the ubiquitin/proteasome system, while PARKIN remains inactive in the cytosol. Otherwise, PINK accumulates and PARKIN initiates autophagy processes [63]. Studies suggest that the deregulated elimination of bioenergetically compromised mitochondria plays an important role in carcinogenesis and tumor progression [64]. Interestingly, PARKIN has been suggested as a p53 targeted gene, and deficiency of PARKIN has been reported to support the switch to the aerobic glycolysis “Warburg effect” [41,65]. Although we did not observe ER stress, the autophagy and mitophagy sensors seem to mutually and inversely control each other in the two cell types. Indeed, increased expression of PARKIN was observed in HepG2 CM, while PINK1, which is a crucial player in mitophagy, did not change (Figure 3). The direct elimination of compromised mitochondria by LC3 A/B also has been reported [64] and found to be increased in HepG2 CM (Figure 4). Inversely, no change was observed for PARKIN (Figure 3) and LC3 A/B II (Figure 4) expression in MG CM; only PINK1 was decreased compared to the control. 

Nrf2 and NF-κB are the two key transcription factors that regulate cellular responses to oxidative stress and inflammation, respectively, and studies suggest that there is functional crosstalk between these two pathways [45,66]. Interestingly, the expression of Nrf2 was found to increase in both HepG2 CM and MG CM most likely as a response to increased oxidative stress rather than to an inflammatory process as there is no variation in NF-κB expression levels (Figure 5). Moreover, the increased expression of Nrf2 might be responsible for the decreased expression of the inflammation marker iNOS, which is widely implicated in both diseases [46,47,48,49]. 

The cell cycle is implicated in both cancer and neurodegenerative diseases. In our study, the expression levels of p53 and p21 were evaluated. As evidenced by the expression levels of the p53 and p21 markers, no cell cycle change is observed in HepG2 and microglia cells grown in conditioned media.

In conclusion, in HepG2 and in MG cells treated with mutually exchanged conditioned media, we observed the activation of cytoprotective mechanisms against any potential pro-oxidant or pro-inflammatory signals present in secretomes. Only HepG2 cells seem to trigger autophagic processes, thus suggesting a cell-specific adaptive response.

Investigations aimed at identifying the components of the secretomes of the two cell types will be carried out in order to suggest potential therapeutic windows for both diseases.

## 4. Materials and Methods

### 4.1. Cell Culture and Preparation of Conditioned Media

Human hepatoblastoma (HepG2) and human microglia (HMC-III) cells were grown in Dulbecco’s modified Eagle’s medium (DMEM) with a high glucose concentration (4.5 g/L), to which 10% fetal bovine serum (FBS), 2 mM L-glutamine, 100 U/mL penicillin, and 100 µg/mL streptomycin were added. Cells were cultured at 37 °C in a water-saturated atmosphere with 5% CO_2_.

The cells were grown separately in 6-well plates at a density of 2.5 × 10^5^/well. After 24 h, 50% of the volume of the culture medium collected from each of the two cell types was centrifuged at 2500 rpm for 5 min. The supernatant was cross-exchanged between HepG2 and HMC-III cells (Cells CM), which were left to grow for 48 h. Control cells (Ctr) continued to grow in their own medium for 48 h. Where indicated, the data of each cell type grown in conditioned medium (CM) refer to the corresponding control cells grown in their own culture medium (Ctr).

### 4.2. Viability Assay

Cell viability was assessed using the MTT (3-(4,5-dimethyl thiazol-2yl)-2,5-diphenyl tetrazolium bromide) assay. In 96-well plates, both cells were seeded separately at a density of 15,000 cells per well in triplicate. After 24 h, the 50 % media were collected and after centrifugation at 2500 rpm at 5 min, supernatant (CM) was cross exchanged between both of the cells for 48 h excluding the control cells. After 48 h, the cells were incubated with 0.75 mg/mL MTT dissolved in fresh DMEM for 4 h at 37 °C. The MTT solution was removed, and cells were treated with 1:1 DMSO and isopropanol with 1% of Triton X-100 to solubilize the formazan crystal at room temperature. The cell viability was assessed by light absorption at 570 nm with a background subtraction at 630 nm of both control and media-exchanged cells using a microplate reader (MultiskanTM GO Microplate Spectrophotometer) (Thermo Scientific, Waltham, MA, USA). The viability of CM cells was determined by comparing absorbance values with their respective control cells (defined as 100% cell viability).

### 4.3. Real-Time PCR

RNA was extracted by using a Quick-RNA MiniPrep kit (ZymoResearch, Irvine, CA, USA) and retrotranscripted to cDNA by using random primers and the High-Capacity cDNA Reverse Transcription kit (Applied Biosystem, Waltham, MA, USA). After retro-transcription, the total cDNA was amplified by using specific primer-designed exon–exon junctions (Table 1) and an iTaqTM Universal SYBR Green Supermix (Bio-Rad, Waltham, MA, USA) with the 7500 Fast Real-Time PCR System (Applied Biosystems).

The relative amount of transcript products was quantified using a comparative threshold cycle method (2^−ΔCt^) considering β-actin as the endogenous reference control. The melting curve was analyzed to confirm amplicon specificity and each test was performed at least in triplicate.

### 4.4. Western Blot Analysis

The western blot analysis was performed as per a previously reported method [67] with some modifications. The cell pellets obtained from HepG2 and HMC-III were suspended by performing gentle pipetting in an appropriate volume of RIPA lysis buffer (Tris-HCl 50 mM pH 8, NaCl 150 mM, NP-40 1%, SDS 0.1%, Sodium deoxycholate 0.5%), protease inhibitor cocktail (PIC), and Phenyl-Methyl-Sulfonyl-Fluoride (PMSF). After sonication, the samples were centrifuged at 13,000 rpm for 10 min at 4 °C. The supernatant was collected and the protein content in each sample was assessed using a Bradford (B6916, Sigma Aldrich, Darmstadt, Germany) colorimetric assay against bovine serum albumin standard (BSA, 23209 Thermo Scientific™, USA) solution by measuring the absorbance at 595 nm with a spectrophotometer (“Multiskan GO”, Thermo Scientific). 

The extracted proteins were resuspended in a sample buffer (375 mM Tris–HCl pH 6.8, 60% glycerol, 12% SDS, 0.6% bromophenol blue, and Dithiothreitol 600 mM), resolved on 12 or 15 % SDS-PAGE gels, and transferred to nitrocellulose membranes. The membranes were blocked for 1 h with a saturation buffer (5% nonfat dried milk or albumin (BSA) in PBS or TBS with 0.05 %Tween 20) and then incubated with primary antibodies overnight at 4 °C, with 1:1000 anti-tubulin (T9026, Sigma-Aldrich, Darmstadt, Germany), 1:5000 anti-GAPDH (10494-1-AP, Proteintech, Rosemont, IL, USA), 1:1000 anti-LC3 A/B (D3U4C, Cell Signalling), 1:1000 anti-IRE1α (PA520189, Invitrogen, Waltham, MA, USA), 1:1000 anti-CHOP(MA1250, Invitrogen), 2.5 μg/mL anti-PARKIN (702785,Invitrogen), 1:1000 anti-PINK1 (D8G3, Cell Signalling), 1:400 anti-p53 (SC-126 Santa Cruz, Santa Cruz, CA, USA), and 1:1000 anti-p21 (MA5-14949, Invitrogen). The membranes were washed three times with PBST or TBST and incubated with the appropriate horseradish peroxidase-conjugated secondary antibodies at room temperature for 1 h, and signals were visualized using the ECL™Western Blotting Detection Reagents (GE Healthcare, Chicago, IL, USA) or Super Signal West Femto Maximum Sensitivity Substrate (Thermo Fisher Scientific) with the Chemidoc TM XRS detection system equipped with Image Lab Software for image acquisition (BioRad, Hercules, CA, USA). Densitometric analysis was performed by using Gel Analyzer 2010 software (Debrecen, Hungary). The protein expression level in control samples was taken as 100%. Each result was expressed as a percentage of the value of the control sample. Each test was repeated three times.

### 4.5. Statistical Analysis

All assays were performed independently at least three times. The standard error of the mean was reported as a measure of variability. Statistical analysis was performed with Student’s *t*-test, using GraphPad Prism software. An alpha level of <0.05 was chosen to define statistical significance.

## Figures and Tables

**Table 1:** List of primers used in this study.

Genes	Forward Primer	Reverse Primer
β-Actina	5′-CCTGGCACCCAGCACAAT-3′	5′-GCCGATCCACACGGAGTACT-3′
CHOP	5′-GTACCTATGTTTCACCTCCTG-3′	5′-TCTCCTTCATGCGCTGCTTTC-3′
IRE1α	5′-AAGAGCCTGCCTTTCTCCCA-3′	5′-ATCTGAACTTCGGCATGGGG-3′
PARKIN	5′-ACTGTGCAGAATTGTGACCT-3′	5′-TTCTGGGGTCGTCGCCTCC-3′
PINK1	5’-CAGTCACTTACAGAAAATCCA-3’	5’-CCTGCCGAGATGTTCCACA-3’
iNOS	5′-TCCAGAAGCAGAATGTGACC-3′	5′-AGCCAAATCCAGTCTGCCG-3′
NF-kB	5′-GATGAAGCATGGAACCATGG-3′	5′-CCCAAAGAGGTTTACAGTGT-3′
TNFR2	5′-GGACTGATTGTGGGTGTGAC-3′	5′-CTTATCGGCAGGCAAGTGAG-3′
p53	5′-TGAATGAGGCCTTGGAACTC-3′	5′-ACTTCAGGTGGCTGGAGTG-3′
p21	5′-CTGTCTTGTACCCTTGTGCCT-3′	5′-CGTTTGGAGTGGTAGAAATCTGTC-3′
Nrf2	5′-AACTACTCCCAGGTTGCCCA-3′	5′CATTGTCATCTACAAACGGGAA-3′

CHOP: C/EBP-homologous protein; IRE1α: inositol-requiring transmembrane kinase endoribonuclease-1α; (PARKIN): Parkin RBR E3 ubiquitin-protein ligase; PINK1: PTEN induced Kinase 1; iNOS: inducible nitric oxide synthase; NF-kB: nuclear factor kappa-light-chain-enhancer of activated B cells; TNFR2: tumor necrosis factor receptor 2; p53, TP53/tumor protein p53; p21: cyclin-dependent kinase inhibitor 1; Nrf2: nuclear factor erythroid 2-related factor 2.

## Data Availability

Not applicable.

## References

[1] Houck A.L., Seddighi S., Driver J.A. (2018). At the crossroads between neurodegeneration and cancer: A review of overlapping biology and its implications. Curr. Aging Sci..

[2] Driver J.A. (2012). Understanding the link between cancer and neurodegeneration. J. Geriatr. Oncol..

[3] Seo J., Park M. (2020). Molecular crosstalk between cancer and neurodegenerative diseases. Cell Mol. Life Sci..

[4] Plun-Favreau H., Lewis P.A., Hardy J., Martins L.M., Wood N.W. (2010). Cancer and neurodegeneration: Between the devil and the deep blue sea. PLoS Genet..

[5] Bajaj A., Driver J.A., Schernhammer E.S. (2010). Parkinson’s disease and cancer risk: A systematic review and meta-analysis. Cancer Causes Control.

[6] Ospina-Romero M., Glymour M.M., Hayes-Larson E., Mayeda E.R., Graff R.E., Brenowitz W.D., Ackley S.F., Witte J.S., Kobayashi L.C. (2020). Association between Alzheimer disease and cancer with evaluation of study biases: A systematic review and meta-analysis. JAMA Netw. Open.

[7] Hang Z., Lei T., Zeng Z., Cai S., Bi W., Du H. (2022). Composition of intestinal flora affects the risk relationship between Alzheimer’s disease/Parkinson’s disease and cancer. Biomed. Pharmacother..

[8] Park J.-H., Kim D.-H., Park Y.-G., Kwon D.-Y., Choi M., Jung J.-H., Han K. (2019). Cancer risk in patients with Parkinson’s disease in South Korea: A nationwide, population-based cohort study. Eur. J. Cancer.

[9] Ibáñez K., Boullosa C., Tabarés-Seisdedos R., Baudot A., Valencia A. (2014). Molecular evidence for the inverse comorbidity between central nervous system disorders and cancers detected by transcriptomic meta-analyses. PLoS Genet..

[10] Forés-Martos J., Boullosa C., Rodrigo-Domínguez D., Sánchez-Valle J., Suay-García B., Climent J., Falcó A., Valencia A., Puig-Butillé J.-A., Puig S. (2021). Transcriptomic and genetic associations between Alzheimer’s disease, Parkinson’s disease, and Cancer. Cancers.

[11] Yalçin M., Malhan D., Basti A., Peralta A.-R., Ferreira J.-J., Relógio A. (2021). A Computational Analysis in a Cohort of Parkinson’s Disease Patients and Clock-Modified Colorectal Cancer Cells Reveals Common Expression Alterations in Clock-Regulated Genes. Cancers.

[12] Rudzińska M., Parodi A., Balakireva A.V., Chepikova O.E., Venanzi F.M., Zamyatnin A.A. (2020). Cellular aging characteristics and their association with age-related disorders. Antioxidants.

[13] Li Z., Zhang Z., Ren Y., Wang Y., Fang J., Yue H., Ma S., Guan F. (2021). Aging and age-related diseases: From mechanisms to therapeutic strategies. Biogerontology.

[14] Staropoli J.F. (2008). Tumorigenesis and neurodegeneration: Two sides of the same coin?. Bioessays.

[15] Varela L., Garcia-Rendueles M.E. (2022). Oncogenic Pathways in Neurodegenerative Diseases. Int. J. Mol. Sci..

[16] Zhao Y., Seluanov A., Gorbunova V. (2021). Revelations about aging and disease from unconventional vertebrate model organisms. Annu. Rev. Genet..

[17] Kennedy S.R., Loeb L.A., Herr A.J. (2012). Somatic mutations in aging, cancer and neurodegeneration. Mechanisms of ageing and development. Mech. Ageing Dev..

[18] Lagoumtzi S.M., Chondrogianni N. (2021). Senolytics and senomorphics: Natural and synthetic therapeutics in the treatment of aging and chronic diseases. Free Radic. Biol. Med..

[19] Si H., Liu D. (2014). Dietary antiaging phytochemicals and mechanisms associated with prolonged survival. J. Nutr. Biochem..

[20] Niccoli T., Partridge L. (2012). Ageing as a risk factor for disease. Curr. Boil..

[21] Rosenberg G.A. (2012). Neurological diseases in relation to the blood–brain barrier. J. Cereb. Blood Flow Metab..

[22] Xiao M., Xiao Z.J., Yang B., Lan Z., Fang F. (2020). Blood-brain barrier: More contributor to disruption of central nervous system homeostasis than victim in neurological disorders. Front. Neurosci..

[23] Takata F., Nakagawa S., Matsumoto J., Dohgu S. (2021). Blood-brain barrier dysfunction amplifies the development of neuroinflammation: Understanding of cellular events in brain microvascular endothelial cells for prevention and treatment of BBB dysfunction. Front. Cell. Neurosci..

[24] Sade D., Shaham-Niv S., Arnon Z.A., Tavassoly O., Gazit E. (2018). Seeding of proteins into amyloid structures by metabolite assemblies may clarify certain unexplained epidemiological associations. Open Biol..

[25] Bartman C.R., TeSlaa T., Rabinowitz J.D. (2021). Quantitative flux analysis in mammals. Nat. Metab..

[26] Ou G.-Y., Lin W.-W., Zhao W.-J. (2021). Neuregulins in neurodegenerative diseases. Front. Aging Neurosci..

[27] Di Santo S., Widmer H.R. (2016). Paracrine factors for neurodegenerative disorders: Special emphasis on Parkinson’s disease. Neural Regen. Res..

[28] Nakajima K., Raz A. (2020). Autocrine motility factor and its receptor expression in musculoskeletal tumors. J. Bone Oncol..

[29] Thomas S.K., Lee J., Beatty G.L. (2020). Paracrine and cell autonomous signalling in pancreatic cancer progression and metastasis. EBioMedicine.

[30] Dowling P., Clynes M. (2011). Conditioned media from cell lines: A complementary model to clinical specimens for the discovery of disease-specific biomarkers. Proteomics.

[31] Song P., Kwon Y., Joo J.-Y., Kim D.-G., Yoon J.-H. (2019). Secretomics to discover regulators in diseases. Int. J. Mol. Sci..

[32] Masuda T., Sankowski R., Staszewski O., Prinz M. (2020). Microglia heterogeneity in the single-cell era. Cell Rep..

[33] Colonna M., Butovsky O. (2017). Microglia function in the central nervous system during health and neurodegeneration. Annu. Rev. Immunol..

[34] Hickman S., Izzy S., Sen P., Morsett L., El Khoury J. (2018). Microglia in neurodegeneration. Nat. Neurosci..

[35] Xu Y., Jin M.-Z., Yang Z.-Y., Jin W.-L. (2021). Microglia in neurodegenerative diseases. Neural Regen. Res..

[36] Lull M.E., Block M.L. (2010). Microglial activation and chronic neurodegeneration. Neurotherapeutics.

[37] Šešelja K., Bazina I., Welss J., Schicht M., Paulsen F., Bijelić N., Rođak E., Horvatić A., Gelemanović A., Mihalj M. (2019). Effect of Tff3 deficiency and ER stress in the liver. Int. J. Mol. Sci..

[38] Kim C., Kim B. (2018). Anti-cancer natural products and their bioactive compounds inducing ER stress-mediated apoptosis: A review. Nutrients.

[39] Sprenkle N.-T., Sims S.-G., Sánchez C.-L., Meares G.-P. (2017). Endoplasmic reticulum stress and inflammation in the central nervous system. Mol. Neurodegener..

[40] Mao L., Liu H., Zhang R., Deng Y., Hao Y., Liao W., Yuan M., Sun S. (2021). PINK1/Parkin-mediated mitophagy inhibits warangalone-induced mitochondrial apoptosis in breast cancer cells. Aging.

[41] Denisenko T.V., Gogvadze V., Zhivotovsky B. (2021). Mitophagy in carcinogenesis and cancer treatment. Discov. Oncol..

[42] Quinn P.M., Moreira P.I., Ambrósio A.F., Alves C.H. (2020). PINK1/PARKIN signalling in neurodegeneration and neuroinflammation. Acta Neuropathol. Commun..

[43] Ge P., Dawson V.L., Dawson T.M. (2020). PINK1 and Parkin mitochondrial quality control: A source of regional vulnerability in Parkinson’s disease. Mol. Neurodegener..

[44] Cui J., Shen H.-M., Lim L.-H.-K. (2020). The role of autophagy in liver cancer: Crosstalk in signaling pathways and potential therapeutic targets. Pharmaceuticals.

[45] Nah J., Yuan J., Jung Y.-K. (2015). Autophagy in neurodegenerative diseases: From mechanism to therapeutic approach. Mol. Cells.

[46] Vannini F., Kashfi K., Nath N. (2015). The dual role of iNOS in cancer. Redox Biol..

[47] Xia Y., Shen S., Verma I.-M. (2014). NF-κB, an active player in human cancers. Cancer Immunol. Res..

[48] Zaghloul N., Kurepa D., Bader M.-Y., Nagy N., Ahmed M.-N. (2020). Prophylactic inhibition of NF-κB expression in microglia leads to attenuation of hypoxic ischemic injury of the immature brain. J. Neuroinflamm..

[49] Saha R.-N., Pahan K. (2006). Regulation of inducible nitric oxide synthase gene in glial cells. Antioxid Redox Signal..

[50] Ahmed S.M.U., Luo L., Namani A., Wang X.J., Tang X. (2017). Nrf2 signaling pathway: Pivotal roles in inflammation. Biochim. Biophys. Acta Mol. Basis Dis..

[51] Esteras N., Dinkova-Kostova A.T., Abramov A.Y. (2016). Nrf2 activation in the treatment of neurodegenerative diseases: A focus on its role in mitochondrial bioenergetics and function. Biol. Chem..

[52] Yang Y., Islam M.-S., Hu Y., Chen X. (2021). TNFR2: Role in cancer immunology and immunotherapy. Immunotargets Ther..

[53] Bai J., Zhang Y., Ding B., Li H. (2022). Targeting TNFR2 in cancer: All roads lead to Rome. Front. Immunol..

[54] Veroni C., Gabriele L., Canini I., Castiello L., Coccia E., Remoli M.-E., Columba-Cabezas S., Aricò E., Aloisi F., Agresti C. (2010). Activation of TNF receptor 2 in microglia promotes induction of anti-inflammatory pathways. Mol. Cell. Neurosci..

[55] Papazian I., Tsoukala E., Boutou A., Karamita M., Kambas K., Iliopoulou L., Fischer R., Kontermann R.E., Denis M.C., Kollias G. (2021). Fundamentally different roles of neuronal TNF receptors in CNS pathology: TNFR1 and IKKβ promote microglial responses and tissue injury in demyelination while TNFR2 protects against excitotoxicity in mice. J. Neuroinflamm..

[56] Jebelli J., Hooper C., Pocock J.-M. (2014). Microglial p53 activation is detrimental to neuronal synapses during activation-induced inflammation: Implications for neurodegeneration. Neurosci. Lett..

[57] Aloi M.-S., Su W., Garden G.-A. (2015). The p53 transcriptional network influences microglia behavior and neuroinflammation. Crit. Rev. Immunol..

[58] Corazzari M., Gagliardi M., Fimia G.M., Piacentini M. (2017). Endoplasmicreticulum stress, unfoldedproteinresponse, and cancercell fate. Front. Oncol..

[59] Zhao L., Ackerman S.L. (2006). Endoplasmic reticulum stress in health and disease. Curr. Opin. Cell Biol..

[60] Riha R., Gupta-Saraf P., Bhanja P., Badkul S., Saha S. (2017). Stressed out–therapeutic implications of ER stress related cancer research. Oncomedicine.

[61] Yadav R.K., Chae S.-W., Kim H.-R., Chae H.J. (2014). Endoplasmic reticulum stress and cancer. J. Cancer Prev..

[62] Lindholm D., Wootz H., Korhonen L. (2006). ER stress and neurodegenerative diseases. Cell Death Differ..

[63] Senft D., Ze’ev A.R. (2015). UPR, autophagy, and mitochondria crosstalk underlies the ER stress response. Trends Biochem. Sci..

[64] Panigrahi D.P., Praharaj P.P., Bhol C.S., Mahapatra K.K., Patra S., Behera B.P., Mishra S.R. (2019). The emerging, multifaceted role of mitophagy in cancer and cancer therapeutics. Semin. Cancer Biol..

[65] Zhang C., Lin M., Wu R., Wang X., Yang B., Levine A.J., Hu W., Feng Z. (2011). Parkin, a p53 target gene, mediates the role of p53 in glucose metabolism and the Warburg effect. Proc. Natl. Acad. Sci. USA.

[66] Wardyn J.D., Ponsford A.H., Sanderson C.M. (2015). Dissecting molecular cross-talk between Nrf2 and NF-κB response pathways. Biochem. Soc. Trans..

[67] Abruzzese V., Matera I., Martinelli F., Carmosino M., Koshal P., Milella L., Bisaccia F., Ostuni A. (2021). Effect of Quercetin on ABCC6 Transporter: Implication in HepG2 Migration. Int. J. Mol. Sci..

